# Weaker control of the electrical properties of cerebellar granule cells by tonically active GABA_A_ receptors in the Ts65Dn mouse model of Down’s syndrome

**DOI:** 10.1186/1756-6606-6-33

**Published:** 2013-07-19

**Authors:** Marianna Szemes, Rachel L Davies, Claire LP Garden, Maria M Usowicz

**Affiliations:** 1 Present address: School of Cellular and Molecular Medicine, University of Bristol, University Walk, Bristol BS8 1TD, UK; 2 Present address: Research & Enterprise Development, University of Bristol, Senate House, Tyndall Avenue, Bristol BS8 1TH, UK; 3 Present address: School of Life, Sport and Social Sciences, Edinburgh Napier University, Sighthill Court, Edinburgh EH11 4BN, UK; 4 School of Physiology & Pharmacology, University of Bristol, University Walk, Bristol BS8 1TD, UK

**Keywords:** Down syndrome, Down’s syndrome, Cerebellum, Ts65Dn, Granule cell, GABA_A_ receptor, Tonic inhibition, Shunting, qPCR, Single-cell RT-PCR

## Abstract

**Background:**

Down’s syndrome (DS) is caused by triplication of all or part of human chromosome 21 and is characterized by a decrease in the overall size of the brain. One of the brain regions most affected is the cerebellum, in which the number of granule cells (GCs) is markedly decreased. GCs process sensory information entering the cerebellum via mossy fibres and pass it on to Purkinje cells and inhibitory interneurons. How GCs transform incoming signals depends on their input–output relationship, which is adjusted by tonically active GABA_A_ receptor channels.

**Results:**

We report that in the Ts65Dn mouse model of DS, in which cerebellar volume and GC number are decreased as in DS, the tonic GABA_A_ receptor current in GCs is smaller than in wild-type mice and is less effective in moderating input resistance and raising the minimum current required for action potential firing. We also find that tonically active GABA_A_ receptors curb the height and broaden the width of action potentials in wild-type GCs but not in Ts65Dn GCs. Single-cell real-time quantitative PCR reveals that these electrical differences are accompanied by decreased expression of the gene encoding the GABA_A_ receptor β3 subunit but not genes coding for some of the other GABA_A_ receptor subunits expressed in GCs (α1, α6, β2 and δ).

**Conclusions:**

Weaker moderation of excitability and action potential waveform in GCs of the Ts65Dn mouse by tonically active GABA_A_ receptors is likely to contribute to atypical transfer of information through the cerebellum. Similar changes may occur in DS.

## Background

Down’s syndrome (DS) occurs in different populations in 1 in 450 to 2200 live births [1]. It is caused by the presence of a third copy of all or part of human chromosome 21 (trisomy Hsa21; OMIM ID: 190685) [2], which results in a range of neurological, behavioural and physical phenotypes that vary in occurrence and expressivity between individuals [3]. Characteristics that all individuals with DS display include a smaller brain, a pronounced decrease in neuron number and distorted neuronal morphology in the cerebellum, hippocampus and cerebral cortex [4]. They also show impaired learning, memory and language, delays in the acquisition of motor skills, poor fine motor skills, altered balance and gait, and unclear speech [4,5]. These changes in brain structure and function are accompanied by altered expression of genes on the triplicated Hsa21 as well as on non-trisomic chromosomes [2,3,6]. How information processing in the DS brain is affected to produce the cognitive and motor deficits is incompletely understood [7]. Recent studies in mouse models of DS have made progress in delineating the modifications in hippocampal synaptic transmission and plasticity that contribute to deficits in specific types of memory [8-19]. In comparison, there have been surprisingly few electrophysiological investigations of cerebellar function in mouse models of DS [20,21].

The cerebellum is a key brain structure in the control of movement. The altered gait, posture, stride length, speech production, acquisition of motor skills and quality of fine motor skills observed in DS implicate cerebellar dysfunction [4,5]. This inference is supported by the finding that in individuals with DS, the volume of the cerebellum and the density of cerebellar GCs are reduced by one third and one quarter respectively, through impaired proliferation of precursor cells [22-28]. Increasing evidence suggests that the cerebellum also plays a role in various cognitive functions [29,30] and that cerebellar dysfunction may contribute to some of the cognitive deficits in DS [5]. As GCs process signals transmitted to the cerebellum by mossy fibres and transmit them to Purkinje cells (PCs) and inhibitory interneurons [31], changes in their number, intrinsic electrical properties or synaptic transmission are likely to distort cerebellar processing.

Postnatal maturation of cerebellar GCs in rodents entails increased expression of specific GABA_A_ receptor (GABA_A_R) subunits and the development of a tonic current generated by repeated opening of extrasynaptic GABA_A_R channels [32-35]. Inhibition of the tonic current with a competitive GABA_A_R antagonist demonstrates that it is caused by the activation of GABA_A_Rs by ambient GABA [32,33,35-37]. The tonic current dampens GC excitability through shunting inhibition and so modulates information flow through the cerebellar cortex [33,35,37]. In this study, we made whole-cell patch-clamp recordings of GCs in the Ts65Dn mouse model of DS to determine whether the properties of the tonically active GABA_A_R channels are modified. We assessed whether their impact on the electrical properties of GCs is altered, as this could contribute to the increased excitability and changed action potential (AP) waveform observed previously in Ts65Dn GCs [21]. We also investigated whether expression of GABA_A_R subunit genes is modified in Ts65Dn GCs, by means of reverse transcription and real-time quantitative PCR (qPCR) of single GCs extracted from slices of cerebellum. The Ts65Dn mouse is the most widely investigated model of DS and is generated by triplication of a region of mouse chromosome 16 (Mmu16), which makes it trisomic for approximately half of the orthologous protein-coding genes and a subset of non-protein coding RNAs located on the long arm of Hsa21 [17,28,38]. The Ts65Dn mouse replicates the drop in GC number and density that typifies DS [26,39,40]. It also shows a decrease in the number of cerebellar PCs and displays morphological abnormalities in PC axons [26,39], changes that may be indicative of alterations in DS [28]. The structural changes in the Ts65Dn cerebellum are accompanied by variable changes in cerebellar expression of genes located in the triplicated region of Mmu16 and on non-trisomic chromosomes [41,42].

We describe previously unknown properties of the tonic current and profile gene expression levels for the major GABA_A_R subunits (α1, α6, β2, β3, γ2 and δ) expressed in wild-type GCs. Our recordings indicate that in the Ts65Dn mouse model of DS, the control of the electrical properties of cerebellar GCs by tonically active GABA_A_Rs is weaker. Single-cell qPCR analyses demonstrate a decrease in expression of the GABA_A_R β3 subunit gene in Ts65Dn GCs, but not of most of the other GABA_A_R subunit genes investigated. These differences are likely to affect information flow through the Ts65Dn cerebellum.

## Results

To investigate if the properties of tonically active GABA_A_Rs in cerebellar GCs are altered in the Ts65Dn mouse model of DS, we made whole-cell patch-clamp recordings from GCs of mature Ts65Dn animals and their euploid (wild-type) littermates, aged between postnatal day (P)40 and P60. We used 10 μM SR95531, a competitive antagonist at GABA_A_Rs, to inhibit GABA_A_Rs activated by ambient GABA. This is expected to block all GABA_A_Rs activated by ambient GABA because 10 μM SR95531 reduces currents evoked by exogenous 10 μM GABA in P7-20 rat GCs by more than 99% [36]. Furthermore, the concentration of GABA surrounding GCs is estimated to be less than 200 nM in P21-40 wild-type rats [43] and more than 80% of the tonic current in these cells is inhibited by 200 nM SR95531 [43]. In previous studies, 10 μM SR95531 has been used to inhibit tonic currents or tonic inhibition in P35-45 rodent GCs under respectively voltage-clamp or current-clamp [35,44]. We also compared expression of genes encoding GABA_A_R subunits by means of single-cell qPCR in P42-69 GCs. The data presented were obtained from slices derived from 68 Ts65Dn mice and 95 wild-type mice.

### Tonic GABA_A_R-mediated current-density is reduced in Ts65Dn GCs

**Figure 1:**
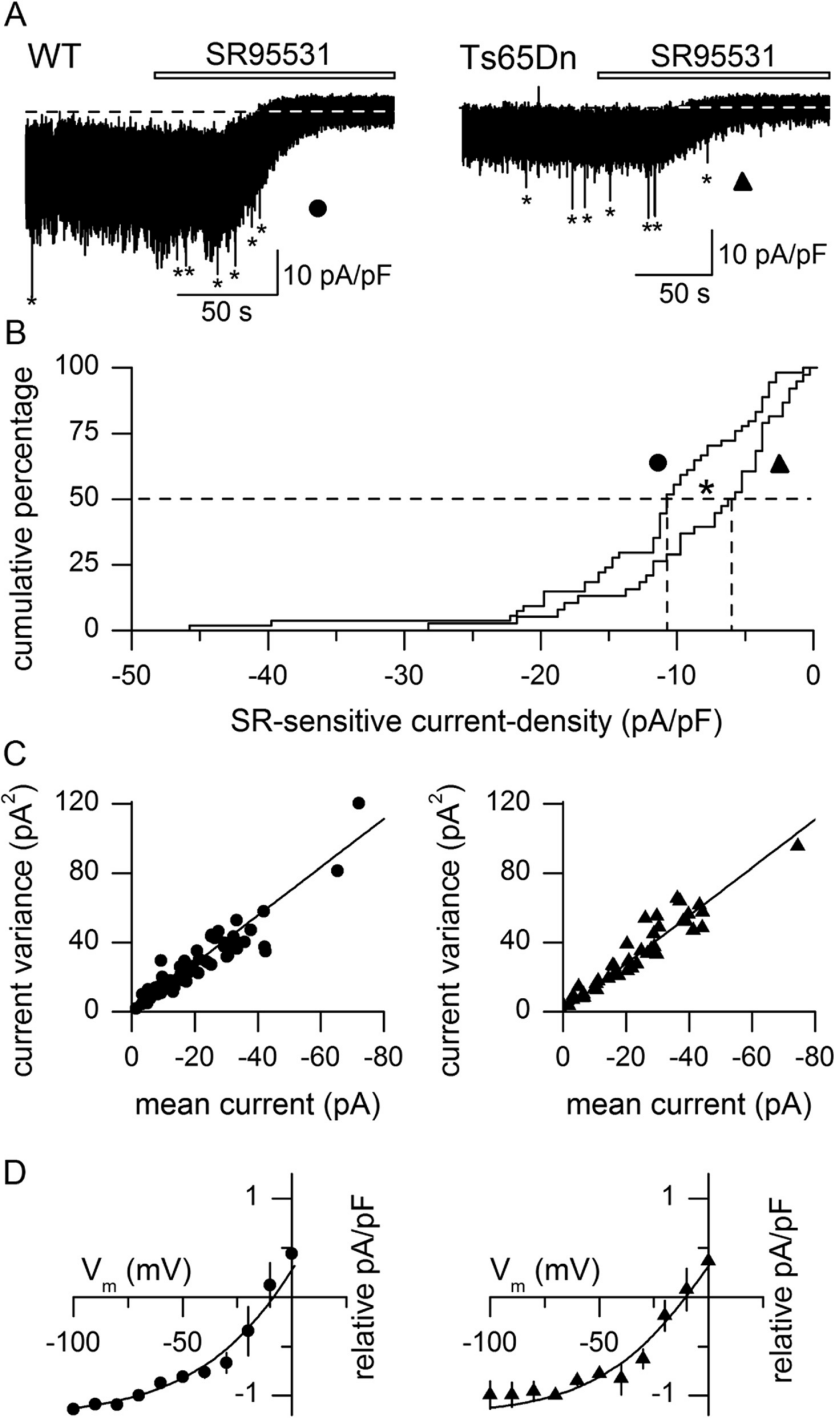
**Tonic GABA**_**A**_**R-mediated current-density is reduced in Ts65Dn cerebellar GCs. (A)** Current recordings from a wild-type GC (WT, filled circle) and a Ts65Dn GC (filled triangle) held at −70 mV, before and during application of SR95531 (10 μM, horizontal bar), with a pipette containing a high Cl^−^ concentration (E_Cl_ ~0 mV). Current amplitudes have been normalised by input capacitance. Dashed lines indicate mean current remaining after block of GABA_A_Rs with SR95531. Asterisks indicate phasic currents. **(B)** Cumulative distribution of tonic GABA_A_R-mediated current-densities in mature wild-type (*n* = 54) and Ts65Dn (*n* = 38) GCs at a holding potential of −70 mV. Dashed lines point to median values (*p = 0.0192, Mann–Whitney *U* test). **(C)** Plots of the variance of the SR-sensitive tonic current against the mean of the SR-sensitive tonic current at a holding potential of −70 mV in wild-type (circles) and Ts65Dn (triangles) GCs. Each point represents a different cell. The line on each plot is drawn through the origin with the same slope of −1.39 pA, which corresponds to the single-channel current, since linear-regression lines fitted to both sets of data did not differ (slope, *p* = 0.1475; intercept, *p* = 0.5354, ANCOVA). **(D)** Plots of current-density (mean ± SEM) against membrane potential, expressed relative to values at −70 mV in wild-type (*n* = 5, circles) and Ts65Dn (*n* = 5, triangles) cells. Solid lines are fitted sigmoidal curves.

Application of SR95531 (10 μM) to cells in voltage-clamp (held at −70 mV with a pipette containing a high Cl^−^ concentration; chloride reversal potential, E_Cl_, ~0 mV) caused a positive shift in mean current and a decrease in the amplitude of current fluctuations (variance) in both wild-type and Ts65Dn GCs (Figure 1A), indicating that GABA_A_R channels are continuously, or tonically, opening in both cell-types. However, the tonic GABA_A_R current-density (current divided by cell input capacitance in order to correct for variation in cell surface area) was significantly lower in Ts65Dn than in wild-type GCs (Figure 1B) (median and quartile values, wild-type, 10.6 pA/pF (14.8, 5.9), *n* = 54; Ts65Dn, 5.8 pA/pF (11.5, 3.8), *n* = 38; equivalent to chord conductances of 172 pS/pF and 97 pS/pF). The reduced tonic current-density did not reflect smaller current flow through individual channels, as there was no difference between wild-type and Ts65Dn GCs in the mean single-channel current amplitude, calculated as the slope of plots of current variance against current mean [45], for whole-cell currents inhibited by SR95531 (Figure 1C, −1.39 pA at −70 mV; corresponds to a mean unitary chord conductance of ~23 pS).

We compared the amplitudes of whole-cell tonic currents inhibited by SR95531 at different membrane potentials in some cells, in order to determine if there was a difference in voltage-dependence of the tonic GABA_A_R current, as such a difference could potentially contribute to different electrical properties of wild-type and Ts65Dn GCs [46]. Figure 1D shows that the dependence of current-density on voltage was non-linear in both wild-type and Ts65Dn GCs; alterations in membrane potential below ~−40 mV caused smaller changes in current-density than changes in membrane potential above ~−40 mV. The slope conductance (calculated from plots of absolute current-density against voltage) increased from 23 ± 14 pS/pF at −100 mV to 283 ± 98 pS/pF at −10 mV in wild-type GCs (*n* = 5), and from 16 ± 8 pS/pF at −100 mV to 216 ± 112 pS/pF at −10 mV in Ts65Dn GCs (*n* = 5). Therefore, the tonic GABA_A_R current showed outward rectification in both wild-type and Ts65Dn GCs.

During postnatal development of wild-type cerebellar GCs, spontaneously-occurring discrete phasic postsynaptic GABAergic currents decrease in size and frequency, while the tonic GABAergic current appears at ~ P7 and increases in magnitude, so that in mature GCs most of the spontaneous GABA_A_R-mediated charge transfer occurs via tonically active GABA_A_Rs [32,33,37]. We found that phasic postsynaptic currents that were identified as GABA_A_R-mediated by their sensitivity to SR95531 also occur at low frequency in Ts65Dn GCs (Figure 1A; median and quartile values, wild-type 0.28 Hz (0.12, 0.75), *n* = 67; Ts65Dn, 0.50 Hz (0.12, 1.01), *n* = 47; *p* = 0.1577, Mann Whitney *U* test) and that there was no change in their mean amplitude (median and quartile values, wild-type, -29.2 pA (−22.7, -35.78), *n* = 59; Ts65Dn, -26.9 pA (−21.2, -37.3), *n* = 41; *p* = 0.8835 ) or decay time course (median and quartile values of weighted decay time constant, wild-type, 7.8 ms (4.4, 10.9); Ts65Dn, 5.9 ms (4.5, 8.0); *p* = 0.1095, Mann Whitney *U* test). They carried a minor percentage of the total charge transferred by spontaneously active GABA_A_Rs, which did not differ from that in wild-type GCs (median and quartile values, wild-type, 3.3% (1.8, 5.0); Ts65Dn, 3.9% (2.0, 6.7), *p* = 0.2068, Mann Whitney *U* test).

### Unchanged relative contributions of δ and α6 subunits to GABA_A_Rs mediating tonic currents in Ts65Dn GCs

**Figure 2:**
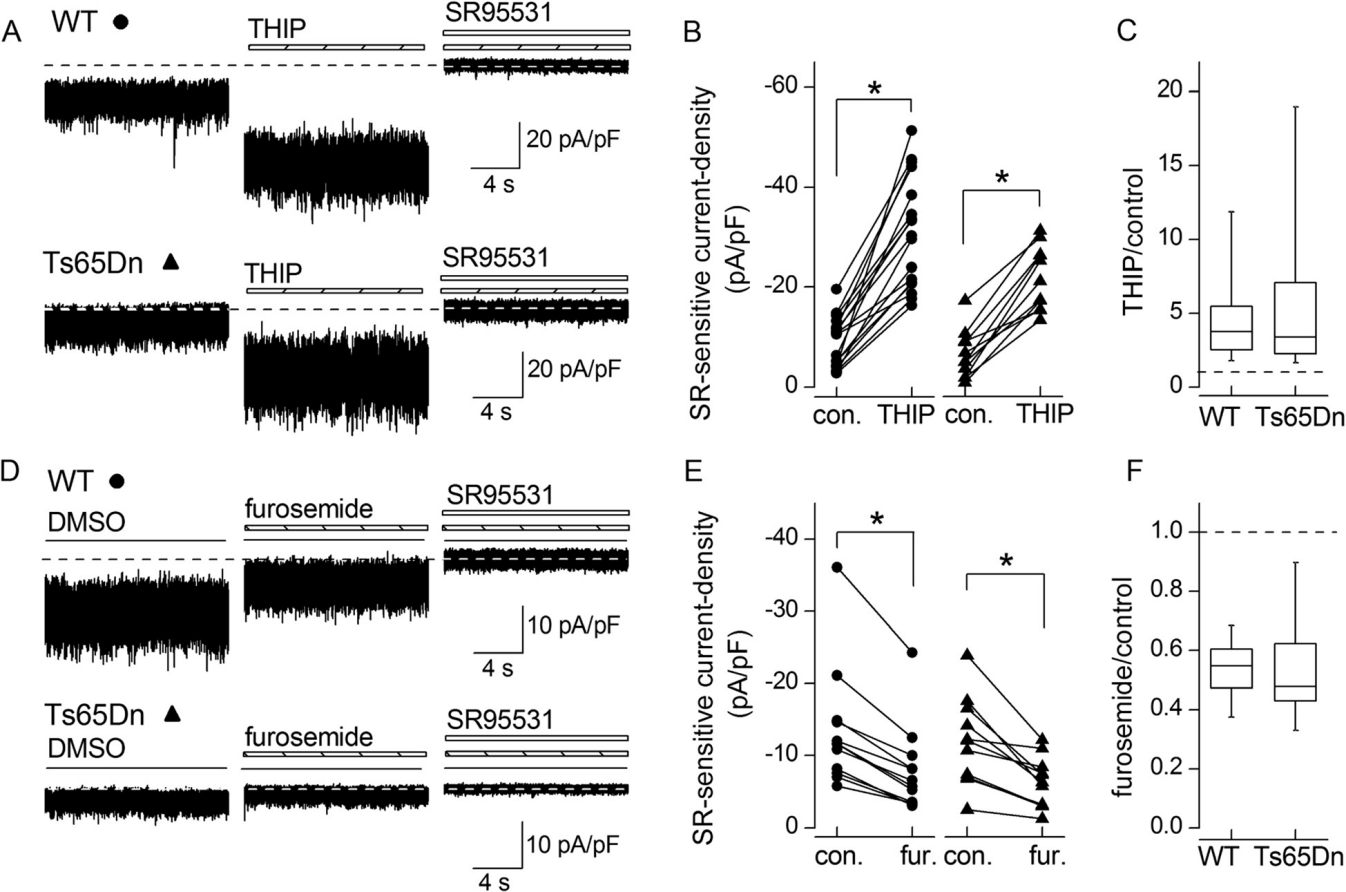
**Unchanged contributions of δ or α6 containing GABA**_**A**_**Rs to the tonic current in Ts65Dn GCs. (A)** Extracts of continuous current recordings from a wild-type GC (WT, filled circle) and a Ts65Dn GC (filled triangle) held at −70 mV under three conditions: before drug application (control), in the presence of the δ-selective agonist, THIP (300 nM, dashed horizontal bar) and in THIP (300 nM) plus SR95531 (10 μM, white horizontal bar). Current amplitude is normalised by cell capacitance. Dashed lines: current remaining after block of GABA_A_Rs with SR95531. The tonic GABA_A_R-current, measured as the difference between currents in the presence and absence of SR95531, was enhanced by THIP. The increase was associated with an increase in noise. **(B)** Summary of tonic GABA_A_R current-densities in the absence (con.) and presence of THIP in individual wild-type (*p < 0.0001, Student’s paired *t* test) and Ts65Dn (*p < 0.0001, Student’s paired *t* test) GCs. **(C)** Box plot of current-densities in THIP expressed relative to control values (dashed line). The enhancement by THIP was not different in wild-type and Ts65Dn cells (*p = 0.9625, Mann–Whitney *U* test). **(D)** Recordings arranged as in A, showing a decrease in tonic current and noise caused by the α6-selective antagonist, furosemide (100 μM, dashed horizontal bars). Control recordings were made in the presence of DMSO (horizontal lines) at a concentration (0.1%) equal to that present during the application of furosemide. **(E)**. Tonic GABA_A_R current-densities in the absence (con.) and presence of furosemide (fur.) in wild-type (*p = 0.0005, Mann Whitney *U* test) and Ts65Dn (*p = 0.0007, Student’s paired *t* test) GCs. **(F)** Box plot of current-densities in furosemide relative to control values (dashed line). The fractional inhibition by furosemide was not different in wild-type and Ts65Dn cells (*p = 0.7520, Mann–Whitney *U* test).

**Figure 3:**
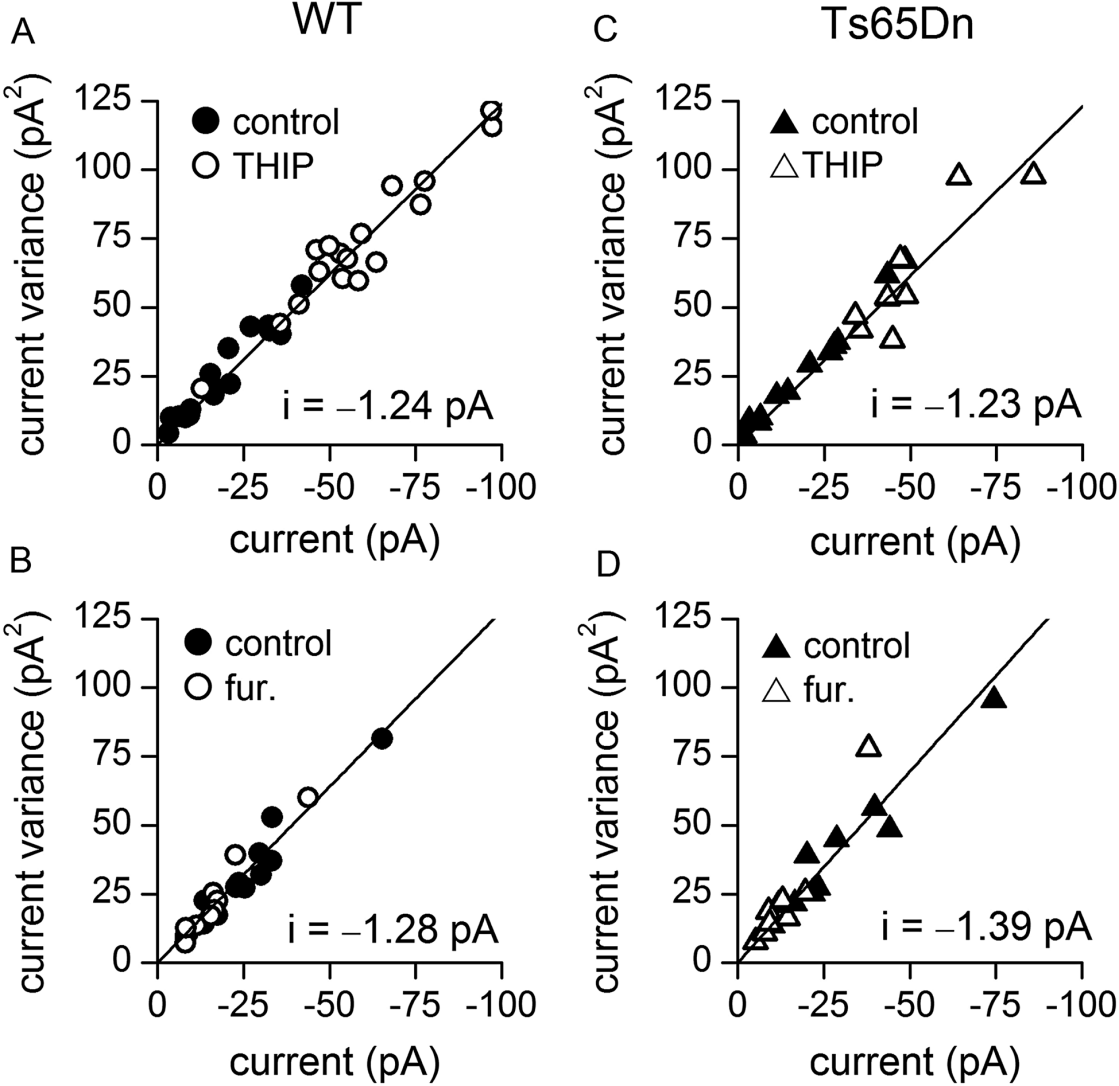
**Enhancement or inhibition of GABA**_**A**_**Rs with subunit-selective drugs reveals no difference in single-channel current amplitude. (A** and **B)** Plots of current variance against mean tonic GABA_A_R currents in wild-type (WT) cells in the absence (filled circles) and presence (empty circles) of 300 nM THIP (A), and 100 μM furosemide **(B**, fur**)**. **(C** and **D)** Same as **(A** and **B)**, but for Ts65Dn GCs (filled and empty triangles). A single least-squares line is fitted to values in the absence and presence of each drug in each plot, as there were no difference between the slopes and y-intercepts of lines fitted to values in the absence or presence of each drug in either wild-type or Ts65Dn GCs (*p* > 0.05 for all comparisons, ANCOVA). The slopes and intercepts of the lines shown did not differ between wild-type and Ts65Dn GCs (slope and intercept: THIP, *p* = 0.8783 and 0.8539; furosemide, *p* = 0.936 and 0.06818; ANCOVA). The single-channel current, i, calculated as the slope of each line, is shown on each plot.

The lower tonic current-density in Ts65Dn GCs could potentially reflect altered subunit composition of the underlying GABA_A_Rs, as subunit composition is a key determinant of the probability of channel opening and the extent and speed of receptor desensitisation upon activation by GABA [47]. In wild-type GCs, the tonic current develops postnatally [32,33] in parallel with increasing expression of δ and α6 subunits in extrasynaptic GABA_A_Rs [34,35]. These receptors have high affinity for GABA and are continually activated by submicromolar ambient GABA [48]. We investigated if the contribution of δ or α6 subunits to extrasynaptic receptors was modified in Ts65Dn GCs, by testing the effects of subunit-selective drugs on the tonic current blocked by SR95531. Figure 2A and 2B show that application of the agonist THIP (gaboxadol), at a δ-selective concentration of 300 nM [49], enhanced the current in both wild-type and Ts65Dn GCs. There was no difference in the magnitude of the effect (Figure 2C). Figure 2D - F show that furosemide, at a concentration (100 μM) at which it is selective for the α6–containing GABA_A_Rs in GCs [50,51], inhibited the tonic current by a similar degree in wild-type and Ts65Dn GCs. Furosemide also binds to α4-containing GABA_A_Rs but with lower affinity [52] and α4-receptors are absent from cerebellar GCs [53]. The changes in the size of the current produced by THIP or furosemide were accompanied by proportionate changes in variance, as the drugs did not alter the linear relationship between SR95531-sensitive variance and mean current (Figure 3). This indicates that there were no differences in mean unitary current amplitude of drug-sensitive and drug-resistant tonically active GABA_A_Rs in Ts65Dn and wild-type GCs. These pharmacological investigations show that the relative contributions of δ and α6 subunit-containing GABA_A_Rs to the tonic current and the unitary conductances of these receptors do not differ between wild-type and Ts65Dn GCs.

### GABA_A_R subunit mRNAs in wild-type and Ts65Dn GCs

**Figure 4:**
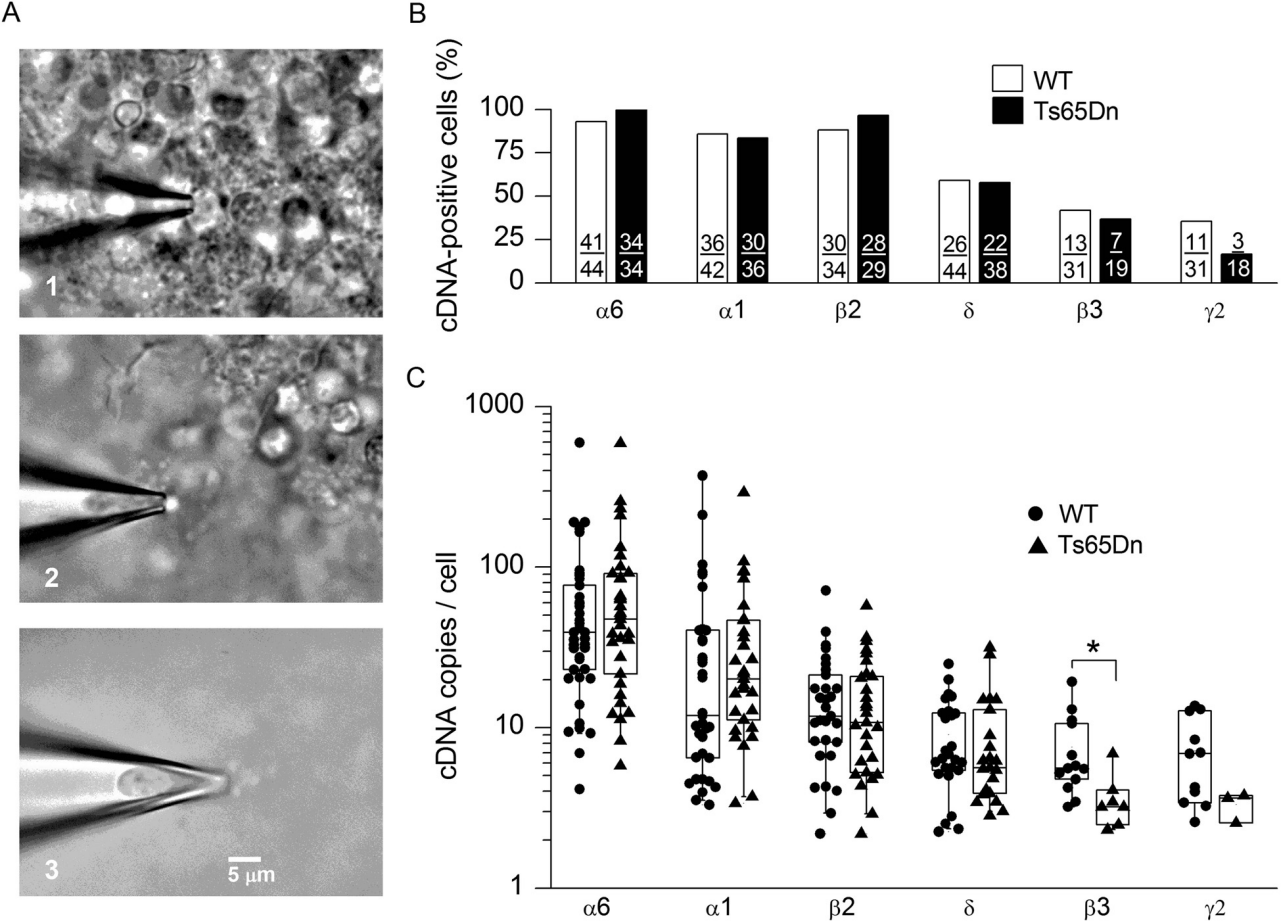
**Comparison of GABA**_**A**_**R subunit gene expression in wild-type and Ts65Dn GCs by single-cell real-time PCR. (A)** Harvesting of a GC into a glass pipette. Calibration bar (5 μm) applies to all panels. 1: pipette tip (containing RNA-protecting solution) touching a chosen cell within a cerebellar slice; 2: suction draws the cell into the pipette. The pipette is raised away from the slice (out of focus) so as to minimise entry of contaminating material; 3: complete cell in the tip, prior to ejection. **(B)** Percentages of wild-type (empty bars) and Ts65Dn (filled bars) GCs in which cDNAs encoding different GABA_A_R subunits (α6, α1, β2, δ, β3 and γ2) were detected. Numbers of cells are shown on the bars. The various cDNAs were detected with dissimilar frequencies in both wild-type (χ^2^(5, n = 226) = 52.73, p < 0.0001) and Ts65Dn (χ^2^(5, n = 174) = 65.83, p < 0.0001) GCs. The frequency with which each subunit cDNA was detected did not differ between wild-type and Ts65Dn (Fisher’s exact test, p > 0.2020 for all comparisons). **(C)** Box plots of numbers of cDNA copies calculated for cDNA-positive GCs (log10 scale). The numbers were not uniform in wild-type (p < 0.0001 Kruskal Wallis test) or Ts65Dn (p < 0.0001 Kruskal Wallis test) GCs but followed the approximate order: α6 ≥ α1 ≥ β2 > δ ≈ β3 ≈ γ2 in both (Dunn’s multiple comparison test p < 0.001 or < 0.01). Copy numbers for α6, α1, β2, and δ cDNA were not different between wild-type and Ts65Dn (p > 0.2800 for all comparisons, Mann Whitney U test), the number of β3 cDNAs was reduced in Ts65Dn (*p = 0.0125, Mann Whitney U test), and the number of Ts65Dn GCs in which γ2 cDNAs were detected was too small for statistical comparison.

To test the possibility that the lower tonic current-density in Ts65Dn GCs was associated with weaker expression of GABA_A_R genes, real-time qPCR was used to quantify GABA_A_R subunit transcripts in individual granule cells. Single-cell mRNA was reverse-transcribed into cDNA and cDNAs encoding δ, α6, β2 and β3 subunits were quantified with real-time PCR, as these encode the extrasynaptic GABA_A_Rs mediating the tonic current in mature wild-type GCs [35,37,43,54-57]. We also measured cDNAs encoding GABA_A_R α1 and γ2 subunits, which together with β2 and β3 subunits form synaptic GABA_A_Rs that mediate phasic currents when transiently activated [54,55,58], because the relative synaptic and extrasynaptic distributions of these subunits are not known for Ts65Dn GCs. Moreover, in wild-type GCs some extrasynaptic receptors may contain α1 subunits and some synaptic receptors contain α6 subunits [43,55,59-62]. For this analysis, individual cells were ‘harvested’ from cerebellar slices into the tip of a blunt pipette, as illustrated in the sequence of photographs in Figure 4A. The pipette tip contained a solution that prevented mRNA degradation during harvesting and processing of the cell prior to reverse-transcription of mRNA into cDNA, all of which were carried out immediately after cell harvesting [63]. The single-cell cDNAs were stored at −20°C until real-time qPCR, which was performed under conditions that had been optimised on GABA_A_R cDNAs generated from wild-type mouse cerebellum (see methods for more details). Only one subunit cDNA was measured per cell.

Figure 4B summarises the percentages of GCs in which expression of a gene encoding one of six GABA_A_R subunits was detected. Considering firstly wild-type GCs, Figure 4B reports wide diversity in the proportions of cells in which the different subunit cDNAs were detected (α6, α1 and β2, 86 - 93% cells; δ, ~59% cells; β3, ~42% cells; γ2, ~35% cells). This denotes disparity in transcription dynamics of GABA_A_R genes, as lower detection rates mirror higher percentages of cells without transcripts at the time of cell sampling. The disparity was also present in Ts65Dn GCs and the detection frequencies for α6, α1, β2, δ and β3 cDNAs in these cells were the same as in wild-type GCs (Figure 4B). Therefore, there were no major alterations to transcription dynamics of α6, α1, β2, δ and β3 subunit genes in Ts65Dn GCs, such as a complete shut down or a several-fold augmentation of transcriptional activity. However, γ2 cDNAs were detected in only half the percentage of Ts65Dn GCs (17%) as in wild-type GCs (35%), although this difference was not statistically different (Fisher’s exact test, *p* = 0.202), possibly because of the relatively small number of Ts65Dn GCs examined for the expression of γ2 mRNA.

The numbers of cDNA copies in the cDNA-positive cells (determined from standard curves, see methods) are summarised in Figure 4C. As expected from the stochastic nature and bursting characteristics of gene transcription [63,64], the values are widely and non-normally distributed. Figure 4C shows marked variation in the numbers of cDNAs encoding different subunits, which parallels the variation in the percentages of cDNA-positive cells shown in Figure 4B. It suggests that relative levels of mRNA expression can be roughly approximated in wild-type GCs by the sequence α6 ≥ α1 ≥ β2 > δ ≈ β3 ≈ γ2. The α6, α1, β2 and δ cDNA copy numbers were not different between wild-type and Ts65Dn GCs, but the β3 cDNA copy number was reduced in Ts65Dn GCs. In addition, the number of γ2 cDNAs appeared to be reduced, but the low number of γ2-positive Ts65Dn GCs precluded a statistical analysis (Figure 4C). These single-cell qPCR data indicate that reduced β3 mRNA expression accompanies the reduced tonic current-density in Ts65Dn GCs. More work is required to confirm the suggested decrease in γ2 mRNA expression.

### Weaker influence of tonic GABA_A_R conductance on input resistance of Ts65Dn GCs

Previous studies have established that a tonic GABA_A_R current dampens the excitability of mature cerebellar GCs because it lowers cell input resistance, which results in smaller changes in voltage in response to current input [35,37]. This shunting inhibition raises rheobase (the minimum current input required to initiate firing of APs) [33,35,37]. Notably, moderation of GC input resistance by tonically active GABA_A_Rs is not accompanied by clear moderation of the resting membrane potential at P18-22 [33] or P30-62 [35,65]. To investigate if shunting inhibition is altered in Ts65Dn GCs, in which we recorded a smaller tonic GABA_A_R current-density in voltage-clamp, we made whole-cell current-clamp recordings before and during block of GABA_A_Rs with 10 μM SR95531. The composition of the pipette solution was similar to that previously used in current-clamp recordings of wild-type cerebellar GCs [44,66,67]. It established an E_Cl_ (~−69 mV) that was more positive than the mean resting potential (~ − 80 mV) of wild-type and Ts65Dn GCs [21], in keeping with the difference between the resting potential and the equilibrium potential for GABA_A_R currents in wild-type GCs measured with the non-invasive perforated patch technique [33,65]. As our voltage-clamp recordings indicated that the charge carried by the tonic current is far in excess of that carried by phasic currents in Ts65Dn GCs as well as in wild-type GCs, the predominant effect of the application of SR95531 on both types of cells in current-clamp is block of tonic inhibition rather than block of phasic inhibition [35,44,67].

**Figure 5:**
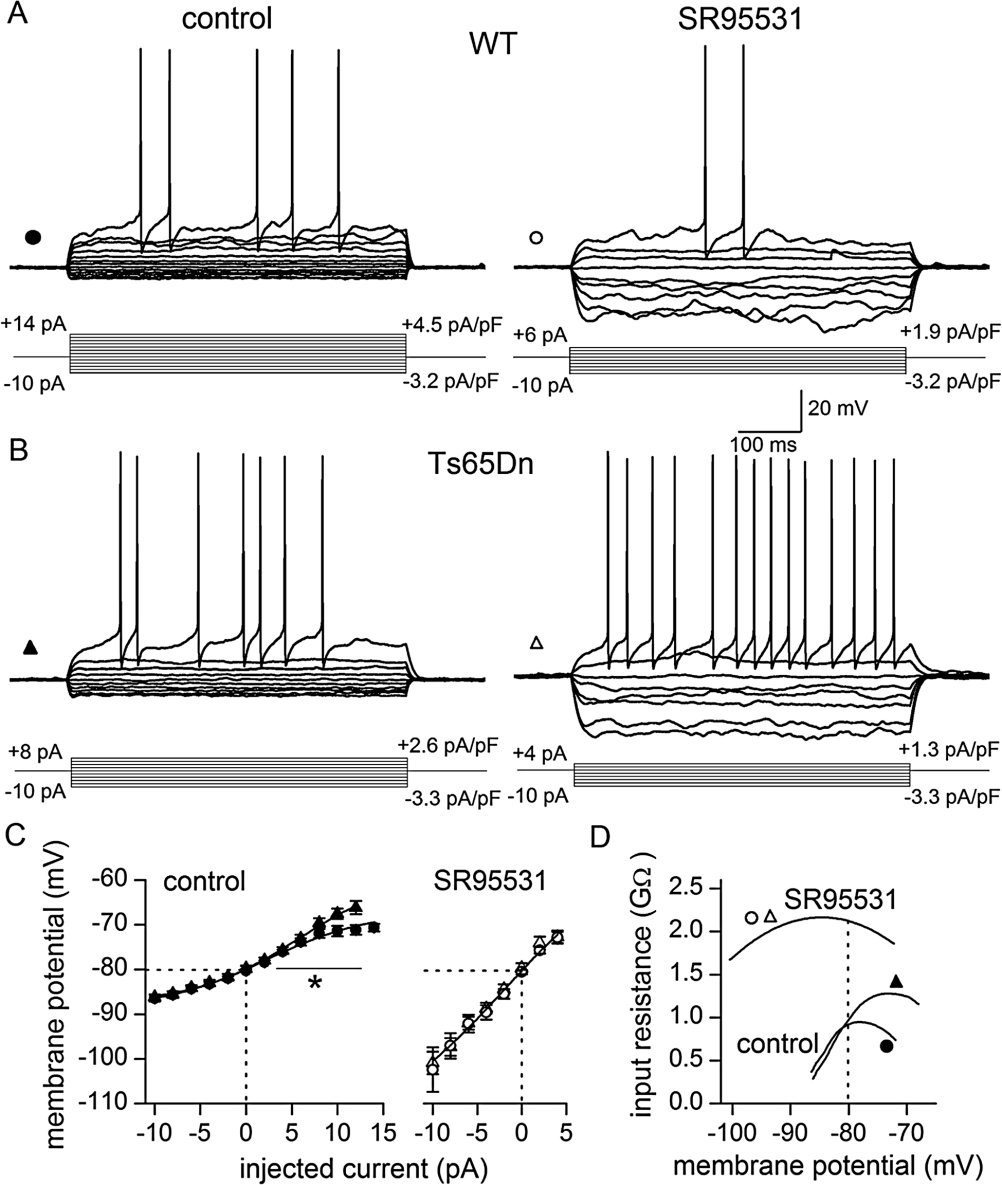
**Moderation of input resistance by tonic activation of GABA**_**A**_**Rs is weaker in Ts65Dn GCs. (A)** Superimposed traces of voltage-recordings during constant current injections (from −10 pA to action potential threshold in +2 pA increments) from a wild-type GC before (filled circle) and after inhibition of tonically-activate GABA_A_Rs with 10 μM SR95531 (empty circle). Current values normalised by input capacitance are also indicated. **(B)** Same as (A), but for a Ts65Dn GC (filled and empty triangles). **(C)** Subthreshold voltage–current relationships (mean ± SEM) for mature wild-type and Ts65Dn GCs before (control) and after application of SR95531 (WT, *n* = 24 – 14; Ts65Dn, *n* = 13 – 8). Dotted lines denote resting membrane potential. Solid lines are fitted sigmoidal curves. In control, the relationships diverged for current injections greater than +2 pA (*f_1_,_27_ = 5.6, *p* = 0.025, two-way repeated measures ANOVA). In SR95531, the relationships were indistinguishable (the same sigmoidal curve is fitted to both sets of data points as an F-test did not reveal differences in the parameters of curves fitted to each data set, F(4,8) = 1.094, *p* = 0.4214). **(D)** Plots of input resistance against membrane potential in control and in the presence of SR95531, obtained by differentiating fitted lines in **(C)**.

Recordings of voltage-changes evoked by current injections showed that before block of tonically active GABA_A_Rs, subthreshold voltage–current relationships were non-linear in both types of cells (Figure 5A - C), but depolarising currents caused greater changes in voltage in Ts65Dn GCs at potentials above resting potential (Figure 5C). Therefore, input resistance changed with voltage in both cell-types but Ts65Dn GCs had higher input resistance at potentials approaching the voltage threshold for AP firing, as reported previously [21]. This difference is clearer in plots of mean input resistance against membrane potential (Figure 5D), derived from the mean voltage–current relationship in Figure 5C. Resting membrane potentials did not differ (Figure 1B, wild-type, −80.21 ± 0.4 mV, *n* = 24; Ts65Dn, −79.76 ± 0.7 mV, *n* = 13; *p* = 0.5137, Student’s unpaired *t*-test). By contrast, after block of tonically active GABA_A_Rs with SR95531, the subthreshold voltage–current relationships in wild-type and Ts65Dn GCs were indistinguishable (Figure 5A - C). Furthermore, they were much steeper indicating that in SR95531 the input resistance was markedly elevated in both cell-types (Figure 5D). In addition, the voltage-dependence of input resistance was weaker (Figure 5D). These changes induced by SR95531 were not accompanied by a change in resting membrane potential in either type of cell (Figure 5C; wild-type: control, −80.21 ± 0.4 mV, SR95531, −80.87 ± 0.8 mV, *n* = 24, *p* = 0.3563 Student’s paired *t*-test; Ts65Dn: control, −79.96 ± 0.7 mV, SR95531, −79.67 ± 1.0 mV, *n* = 13; *p* = 0.9047 Student’s paired *t*-test). Our finding that the impact of the tonic inhibition was weaker on the current–voltage relationship of the Ts65Dn GCs while its lack of effect on the resting potential was unaltered, lends further support to other observations that the predominant effect of tonically active GABA_A_Rs in mature GCs is shunting inhibition rather than alteration of membrane potential [33,35,37]. The lack of a clear effect on the resting potential is thought to reflect the small magnitude of the outward driving force on the Cl^−^ ions [33,65].

The transformations of the subthreshold voltage–current relationships revealed that the tonic GABA_A_R conductance lowers input resistance in a voltage-dependent manner in both wild-type and Ts65Dn GCs, but this decrease was weaker in Ts65Dn GCs at potentials approaching the voltage-threshold for AP firing. The vertical differences between the curves in Figure 5D show that this effect accounts for a mean decrease of ~1.6 GΩ at −85 mV and ~1.1 GΩ at −75 mV in wild-type GCs, and ~1.7 GΩ at −85 mV and ~0.7 GΩ at −75 mV in Ts65Dn GCs. Elimination of the difference between the subthreshold voltage–current relationships of wild-type and Ts65Dn GCs in SR95531 (Figure 5C) demonstrates that the higher input resistance of Ts65Dn GCs at membrane potentials approaching AP threshold under control conditions (Figure 5D) is due to weaker moderation of input resistance by the tonic GABA_A_R conductance. This is consistent with the smaller tonic current-density recorded in Ts65Dn GCs in voltage-clamp.

### Weaker inhibition of Ts65Dn GC excitability by tonic activation of GABA_A_Rs

**Figure 6:**
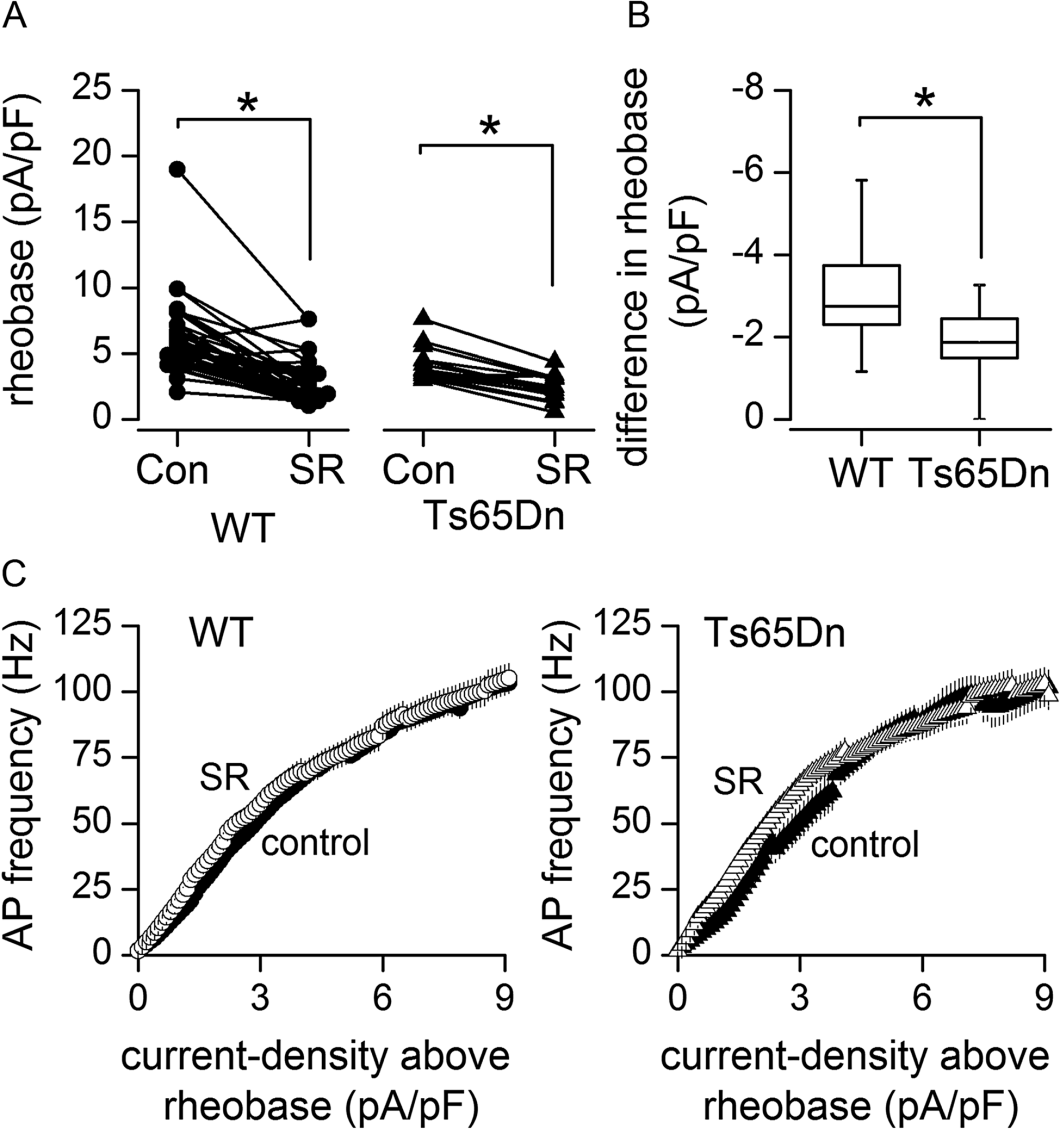
**Tonic activation of GABA**_**A**_**Rs has a weaker impact on excitability of Ts65Dn cerebellar granule cells. (A)** Values of rheobase (minimum current-density required to initiate AP firing) before and during block of GABA_A_Rs with SR95531, in wild-type (*p < 0.0001, Wilcoxon matched pairs test; *n* = 24) and Ts65Dn (*p = 0.0005, Wilcoxon matched pairs test; *n* = 13) GCs. Overlapping points are horizontally offset. **(B)** Box plots comparing the change in rheobase induced by the application of SR95531 in wild-type and Ts65Dn GCs (*p = 0.0145, Mann Whitney *U* test). **(C)** Dependence of AP frequency (mean ± SEM) on magnitude of injected current-density above rheobase in the absence (filled symbols) and presence (empty symbols) of SR95531, in wild-type and Ts65Dn GCs (WT, *n* = 24 – 12; Ts65Dn, *n* = 13 – 5).

In agreement with previous studies of wild-type GCs [35,37,67], the increase in input resistance caused by blockade of GABA_A_Rs with SR95531 was accompanied by a decrease in rheobase (minimum current input required to trigger AP firing) in wild-type GCs. This is illustrated by voltage recordings in Figure 5A and plots summarising rheobase values in Figure 6A. In Ts65Dn GCs, block of GABA_A_Rs also reduced rheobase (Figure 5B and 6A), but the decrease was smaller (Figure 6B), in parallel with the smaller increase in input resistance near AP threshold (Figure 5D). Furthermore, after block of GABA_A_Rs, there was no difference between the rheobase of wild-type and Ts65Dn GCs (median and quartile values, wild-type, 1.9 (1.6, 3.4) pA/pF, *n* = 24; Ts65Dn, 2.4 (1.6, 3.1) pA/pF, *n* = 13, *p* = 0.8056, Mann Whitney *U* test), whereas before the application of SR95531, rheobase was lower in Ts65Dn GCs (wild-type, 5.0 (4.1, 6.5) pA/pF, *n* = 24; Ts65Dn, 3.8 (3.3, 5) pA/pF, *n* = 13, *p* = 0.0211, Mann Whitney *U* test) [21]. The decrease in rheobase upon application of SR95531 was not accompanied by a change in the sensitivity of cell firing to changes in suprathreshold current input in either wild-type or Ts65Dn mice (Figure 6C). It was also not accompanied by changes in latency to first AP at rheobase (wild-type, *p* = 0.3716, Student’s paired *t* test; *n* = 24; Ts65Dn, *p* = 0.2922, Student’s paired *t* test; *n* = 13), which did not differ between wild-type and Ts65Dn before (*p* = 0.9700, Student’s unpaired *t* test) or after (*p* = 0.1358, Student’s unpaired *t* test) application of SR95531. In summary, our finding that rheobase was lower in Ts65Dn than in wild-type GCs before but not after block of GABA_A_Rs, demonstrates that the tonic conductance exerts weaker inhibition of cell-excitability in Ts65Dn GCs.

### Weaker control of action potential waveform in Ts65Dn GCs by tonic activation of GABA_A_Rs

**Figure 7:**
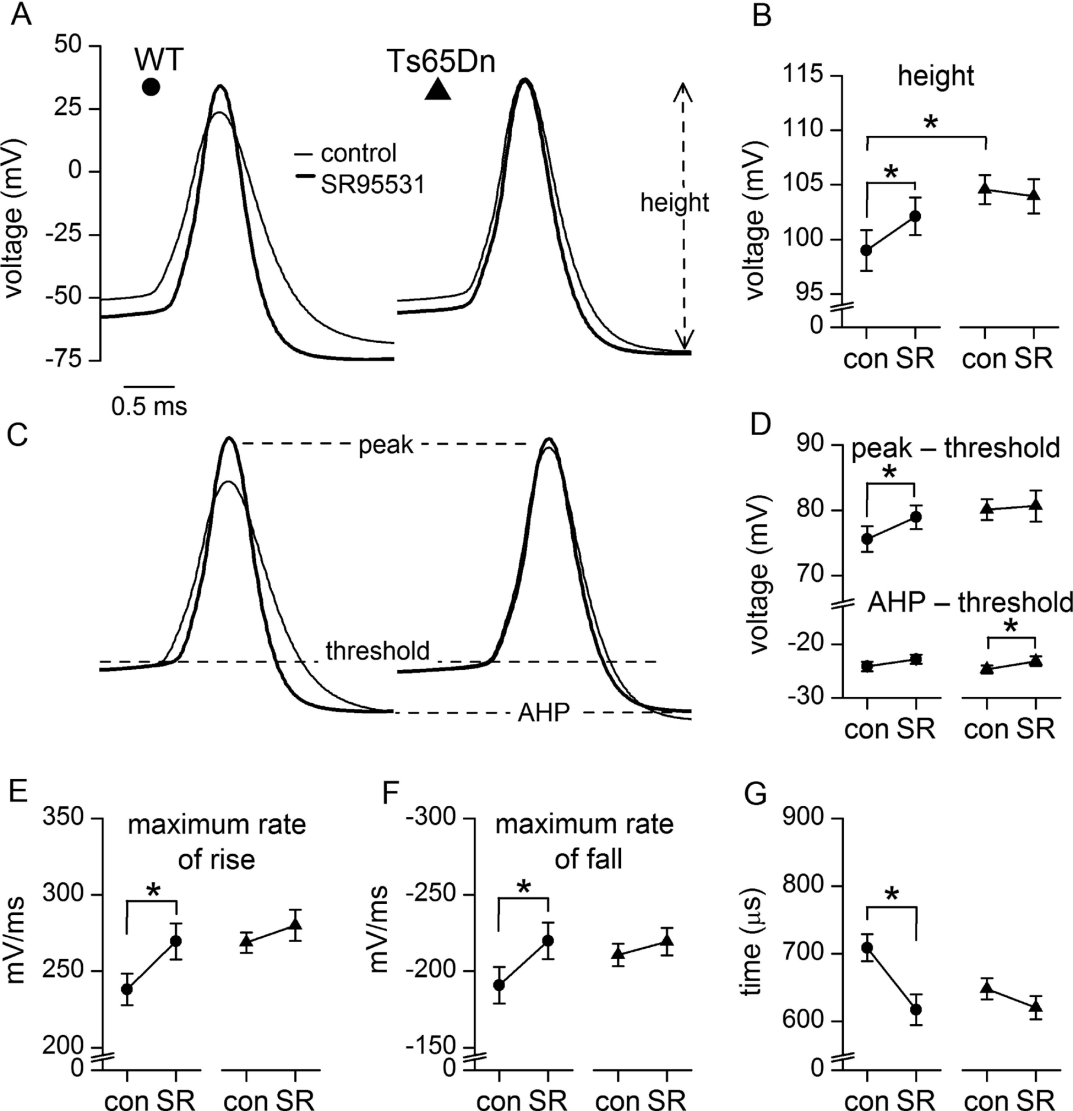
**Tonically-active GABA**_**A**_**Rs modify AP shape more strongly in wild-type GCs than in Ts65Dn GCs. (A)** Superimposed APs (centred on their peak) from a wild-type (filled circle) and a Ts65Dn (filled triangle) GC before (thin line, control) and during inhibition of GABA_A_Rs with 10 μM SR95531 (thick line). Each trace is the average of 3 APs recorded at rheobase. **(B)** Under control conditions (con) AP height (dashed arrow, mean ± SEM) was greater in Ts65Dn GCs (*p = 0.0360, Student’s unpaired t test). SR95531 (SR) increased AP amplitude in wild-type (*p = 0.0125, Student’s paired t test; n = 24) but not in Ts65Dn GCs (p = 0.6553, Student’s paired t test; n = 13); in the presence of SR95531 AP heights in wild-type and Ts65Dn GCs were no longer different (*p = 0.2754, Student’s unpaired t test). **(C)** Traces in **(A)** vertically aligned on their threshold so as to aid shape comparison. **(D)** AP peak and AHP (afterhyperpolarisation) measured from threshold (mean ± SEM) before (con) and during application of SR95531 (SR). SR95531 increased the peak in wild-type (*p = 0.0167, Student’s t paired test) but not in Ts65Dn GCs (p = 0.7074, Student’s paired t test). SR95531 caused small decreases in AHP (wild-type, p = 0.0584; Ts65Dn, *p = 0.0107; Student’s paired t tests). **(E)** Maximum rate of AP rise (mean ± SEM) was speeded up by SR95531 in wild-type (*p = 0.00003, Student’s paired t test) but not Ts65Dn GCs (p = 0.1577, Student’s paired t test). **(F)** As in **(E)**, but comparing maximum rates of fall (WT, *p = 0.0013; Ts65Dn, *p = 0.3116; Student’s paired t tests). **(G)** SR95531 shortened AP width in wild-type (*p = 0.0001, Student’s paired t test) but not in Ts65Dn GCs (p = 0.0132, Student’s paired t test).

Under control conditions, the height of APs (measured between peak and afterhyperpolarisation) was greater in Ts65Dn GCs than in wild-type GCs (Figure 7A and B) [21]. We investigated the possibility that this was also due to a weaker influence of the tonically active GABA_A_Rs in Ts65Dn GCs, although previous studies in wild-type GCs had not reported a moderating effect on AP waveform [35,37]. We found that block of GABA_A_Rs with SR95531 increased AP height in wild-type (by ~4%) but not Ts65Dn GCs, thus eliminating the difference in height present in control conditions (Figure 7A and B). SR95531 also hyperpolarised AP voltage threshold, as illustrated by the vertical displacement of the superimposed traces in Figure 7A (from −47.93 ± 0.63 mV to −52.34 ± 0.66 mV in 24 wild-type GCs, *p* < 0.0001; from −46.51 ± 0.69 mV to −50.13 ± 0.92 mV in 13 Ts65Dn GCs, *p* = 0.0001, Student’s paired *t* tests). This negative shift was similar in magnitude in wild-type and Ts65Dn GCs (wild-type, 4.41 ± 0.64 mV; Ts65Dn GCs, 3.62 ± 0.49 mV, *p* = 0.4040, Student’s unpaired *t* test) unlike the differential effect of SR95531 on AP height. Vertical alignment of APs recorded before and during application of SR95531 on their voltage-thresholds (Figure 7C) revealed that the increase in AP height in wild-type GCs was mainly due to an increase in peak amplitude relative to threshold, which was unchanged in Ts65Dn GCs (Figure 7D). In addition, there was a small decrease in afterhyperpolarisation relative to threshold (wild-type, 1.3 ± 0.7 mV, *n* = 24; Ts65Dn GCs, 1.5 ± 0.5 mV, *n* = 13) that did not differ between cell-type (Figure 7D, *p* = 0.8514, Student’s unpaired *t* test). As well as increasing AP height in wild-type GCs, block of GABA_A_Rs accelerated maximum rates of rise and fall of APs (Figure 7E and F) by a similar percentage (~16 and ~18%, *p* = 0.7156, Student’s unpaired *t* test). This resulted in a shortening of AP duration (Figure 7G) and elimination of the difference in AP width between wild-type and Ts65Dn GCs under control conditions (control: *p* = 0.0473, *n* = 24; SR95531: *p* = 0.9334, *n* = 13, Student’s unpaired *t* test). In contrast, the kinetics and duration of APs in Ts65Dn GCs were not significantly altered in SR95531 (Figure 7E - G). Therefore, tonically active GABA_A_Rs modulate AP waveform in wild-type GCs but have a much weaker effect on AP waveform in Ts65Dn GCs.

Our finding that the changes in AP waveform in wild-type GCs upon inhibition of GABA_A_Rs occurred in the absence of a change in mean resting potential does not support the possibility that the increase in amplitude and narrowing of the APs reflects removal of inactivation of voltage-gated ion channels by reversal of GABA_A_R-induced membrane depolarisation. However, it was possible that a relationship between a change in AP waveform and hyperpolarisation was obscured in our comparison of average values, which masked variation in the effect of SR95531 in different cells between a small decrease, no effect or a small increase in resting potential or AP amplitude. Therefore, we plotted the change in AP amplitude against the change in resting potential for individual wild-type cells. This revealed a weak correlation (*r*(22) = −0.317, *p* = 0.1494), suggesting that a change in membrane potential is unlikely to be the main factor underlying the increase and narrowing of APs caused by inhibition of tonically active GABA_A_Rs.

## Discussion

Our study demonstrates that in wild-type cerebellar GCs, tonic activation of GABA_A_Rs moderates input resistance in a voltage-dependent manner and modifies the waveform of APs. Our study also profiles wide variation in expression of genes encoding α1, α6, δ, γ2, β2 and β3 subunits of GABA_A_Rs in wild-type GCs. In GCs of the Ts65Dn mouse model of DS, tonically active GABA_A_Rs exert a weaker control over input resistance and AP waveform, which results in increased excitability and the firing of APs with increased amplitude. These differences in electrical properties are accompanied by decreased transcription of the Gabrb3 gene encoding the GABA_A_R β3 subunit, in the absence of changes in expression of genes encoding GABA_A_R α1, α6, δ or β2 subunits, while a suggested difference in γ2 gene expression deserves further exploration.

### Tonically active GABA_A_Rs moderate input resistance and action potential waveform

We confirmed that tonically active GABA_A_Rs control input resistance and rheobase in mature wild-type GCs [35,37,67] without a detectable effect on resting membrane potential, as reported previously for mature GCs [35,65], but also uncovered previously unknown characteristics. We find that the tonic GABA_A_R conductance is not constant, as sometimes assumed, but increases in size at voltages approaching threshold, like the outwardly rectifying tonic GABA_A_R current in hippocampal CA1 pyramidal neurons [46]. We also find that tonic activation of GABA_A_Rs slows the kinetics and curbs the height of APs in wild-type GCs. In contrast, tonic activation of GABA_A_Rs has minimal effect on AP shape in Ts65Dn GCs, and APs in wild-type and Ts65Dn GCs are the same height after block of GABA_A_Rs. Our finding that SR95531 had almost no impact on AP waveform in Ts65Dn GCs counteracts the possibility that the effects of SR95531 on APs in wild-type GCs reflect off-target binding to the ion channels that generate the AP (unless these are different in wild-type and Ts65Dn GCs). Further evidence that the effects of SR95531 are mediated by inhibition of GABA_A_Rs, without effects on other channels, is the previously reported lack of an effect of SR95531 on input resistance and rheobase in cerebellar GCs lacking extrasynaptic GABA_A_Rs [35].

It is not clear how tonic activation of GABA_A_Rs modifies AP waveform in wild-type GCs. In hippocampal mossy fibre boutons, GABA_A_Rs tonically activated by ambient GABA also reduce AP amplitude and make APs wider [68]. These changes are thought to reflect enhanced inactivation of voltage-gated sodium channels secondary to depolarisation of the membrane by tonically active GABA_A_Rs [68]. However, such a mechanism seems unlikely to account for the effects of tonically active GABA_A_Rs on APs in wild-type GCs, because the increase in AP amplitude and decrease in AP duration upon inhibition of GABA_A_Rs with SR95531 were not accompanied by detectable changes in membrane potential or in the duration of the subthreshold depolarisation preceding the AP (which might change the fraction of inactivated voltage-gated channels). An alternative explanation is that the outwardly-rectifying tonic conductance generated by the repeatedly opening GABA_A_R channels is able to shunt the currents that generate the AP. Shunting of APs by a GABA_A_R conductance has been previously observed in primary afferents [69] and in the soma of dentate granule neurons [70], and has been demonstrated in a modelling study [71].

### Decreased expression of Gabrb3 in Ts65Dn GCs

Our results indicate that the smaller tonic GABA_A_R current in GCs of Ts65Dn mice does not arise because of a reduced single-channel conductance or differences in the relative contributions of α6 and δ subunits, which are important determinants of GABA potency and receptor desensitisation [43,47,56,72,73]. A contributing factor to the smaller current might be the decreased transcription of Gabrb3 that we detected with single cell qPCR. This could result in fewer receptors at the cell surface because β3 subunits control trafficking of GABA_A_Rs [74]. This possibility has yet to be explored experimentally by measurement of cell surface expression of the receptors or currents evoked by exogenous GABA. Further contributing factors to the smaller tonic current in Ts65Dn GCs may be changes in ambient GABA concentration. A decrease would result in the activation of fewer channels while an increase might also reduce the mean number of channels open if it enhances desensitisation. The GABA concentration is reported to be reduced in foetal DS brains [75] but unchanged in post-mortem samples of cerebellum from adults with DS [76].

Decreased expression of GABA_A_R β3 mRNA or β2/3 protein has been detected before in adult Ts65Dn mouse brain, in the fascia dentata of Ts65Dn hippocampus at 3 months but not at 8 months, in DS brain cells (cultured neural progentior cells derived from foetal DS cortex) but not in the hippocampus of Ts65Dn mice at P15 [13,77-79]. Why transcription of Gabrb3 might be down-regulated is unclear. This cannot be due to a change in gene dosage because Gabrb3 is located on Mmu7 and not on the partially trisomic Mmu16. Likewise, in humans, Gabrb3 is located on Hsa15 and not on Hsa21, which is trisomic in DS. It is possible that in DS, disomic Gabrb3 is modified by overexpressed trisomic microRNAs (miRNAs) or transcription factors located on Hsa21 [2,3,28,80-82]. The trisomic region of Mmu16 in Ts65Dn mice harbours two of the miRNAs located on Hsa21, mir-155 and mir-802, [2,80,81,83,84] and these are overexpressed in Ts65Dn hippocampus and prefrontal cortex [80]. However, they are not predicted to target human or mouse Gabrb3 mRNAs [85-87]. Gabrb3 expression might also be modified by disomic miRNAs or transcription factors, whose expression is altered by trisomic miRNAs or transcription factors or whose properties are postranslationally modified by interactions with trisomic proteins [2,6,78,80-82]. A further possibility is that downregulation of Gabrb3 expression in Ts65Dn GCs is secondary to changes in the ambient concentration of GABA.

### GABAergic transmission is altered in Ts65Dn mice

The smaller tonic GABA_A_R current and its weaker effects in Ts65Dn GCs are in contrast with augmented GABAergic inhibition in dentate granule cells and CA1 pyramidal neurons of Ts65Dn hippocampus [8,10-18,88], which like the cerebellum is markedly reduced in volume and cell-number in individuals with DS and in Ts65Dn mice [4,5,17,28,89]. The enhanced inhibition impairs long term potentiation (LTP) and learning and memory in Ts65Dn mice and is suggested to be due to up regulation of fast or slow phasic transmission by, respectively, GABA_A_ or GABA_B_ receptors [8-18,88]. The increase in GABA_A_R-mediated synaptic transmission is cell and age specific. It does not occur in all subtypes of CA1 neurons or at all ages [13,90], whereas hippocampal CA3 pyramidal neurons of P13-16 Ts65Dn mice show a reduction in inhibitory GABA_A_R-mediated synaptic input and no impairment of LTP [91]. The molecular basis of the enhanced GABA_B_R-mediated inhibition is cell-type specific over expression of the GABA_B_ effector, GIRK2, which occurs because the encoding Kcnj6 gene is triplicated in Ts65Dn mice, as it is in individuals with DS [13,14,92]. In adult Ts65Dn cerebellum, GIRK2 expression is elevated in unipolar brush cells rather than GCs [93]. Our study did not detect differences in the frequency or time course of spontaneous phasic GABA_A_R-mediated postsynaptic currents in cerebellar GCs, but evoked phasic GABA_A_R-mediated transmission has yet to be investigated.

The possibility that increased inhibition in Ts65Dn CA1 hippocampal neurons is due not only to changes in phasic inhibition but also to upregulation of tonic GABA_A_R currents has not been addressed directly, by measuring the tonic GABA_A_R currents that are known to be expressed in these and other types of hippocampal cells [46,56,94-97]. Indirect evidence for their augmentation in the Ts65Dn mouse model of DS is alleviation of cognitive deficits by a selective inverse agonist (α5IA) [98] or a negative allosteric modulator (RO4938581) [99] of α5-containing GABA_A_Rs, which is the class of GABA_A_R that generates a tonic current in hippocampal CA1 and CA3 pyramidal neurons [96,97]. It has also been suggested that the beneficial effects of chronic treatment with α5IA involve changes in gene expression [100].

### Significance of weaker control of electrical properties in Ts65Dn cerebellar GCs by tonically active GABA_A_Rs

The weaker moderation of excitability and AP waveform by tonically active GABA_A_Rs in Ts65Dn GCs predicts that detection and transfer of incoming signals from mossy fibres and hence sensory information processing by the cerebellum is not the same as in wild-type mice. *In vivo* recordings from GCs of young rats (P18-22) show that block of tonically active GABA_A_Rs increases firing of GCs in response to incoming sensory signals. The increase is moderate relative to the greater increase in spontaneous firing [44,67] and hence sensory signals are less likely to be discriminated from the enhanced background activity, resulting in impaired fidelity of sensory transmission through the GC layer. However, it is uncertain how closely these observations predict the effect of tonically active GABA_A_Rs on adult GCs, because the electrical properties of P18-22 and mature GCs are not identical [21,35,44,65,67]. Information transfer by Ts65Dn GCs could also be affected by the change in AP shape, as it is becoming apparent that neural information is encoded not only in the frequency and pattern of firing but also in the shape of APs [101]. For example, the altered AP waveform could distort the time course of glutamate release from GC axons on to Purkinje cell dendrites and inhibitory interneurons. If the changes in GC excitability and AP waveform also occur in DS, reinstatement of the tonic current to an optimal level with agonists or positive allosteric modulators selective for the underlying α6-containing GABA_A_Rs may help correct cerebellar dysfunction. Such drugs should not exacerbate the cognitive deficits in Ts65Dn mice arising from increased inhibition in the hippocampus because the hippocampus, like most brain structures external to the cerebellum, is devoid of α6 GABA_A_Rs [53,57]. On the other hand, if the decrease in tonic current is a compensatory mechanism for the fall in GC number that aims to maintain information flow through the cerebellum, drugs that enhance the function of α6-containing GABA_A_Rs may exacerbate cerebellar dysfunction in DS, since augmentation of tonic inhibition above an optimum level can also impair transfer of sensory transmission through the GC layer of the cerebellar cortex [44].

Drugs that inhibit GABA_A_R function have been suggested as potential treatments for the improvement of mental capacity in DS because they counteract excess inhibition in the hippocampus [5,11,15-17,38,88,98,99]. Since we find that the tonic GABA_A_R current is decreased in cerebellar GCs of the Ts65Dn mouse, such drugs should ideally target GABA_A_Rs in the hippocampus without affecting extrasynaptic GABA_A_Rs in cerebellar GCs. Examples of such drugs are the α5-selective compounds α5IA [98] and RO4938581 [99], because α5-containing GABA_A_Rs are highly expressed in the hippocampus but not the cerebellum [53,57]. In contrast, the interaction of the antagonist pentylenetetrazole with a broader range of GABA_A_Rs may explain why it not only improves memory function in Ts65Dn mice but may also exacerbate their impaired ability to maintain equilibrium, a behaviour that requires the proper operation of the cerebellum [88].

The effects of upregulating the tonic current in cerebellar GCs with drugs selective for α6- containing GABA_A_Rs, could be investigated on cognitive deficits in Ts65Dn mice with standard tests of learning and memory [17,38]. Evaluation of the potential effects of these drugs on speech and language, both of which rely on the correct operation of the cerebellum and are markedly affected in DS [5,30,102,103], might be possible in Ts65Dn mice through the analysis of ultrasonic vocalisations [104], the development of which is delayed in Ts65Dn mice [105]. However, appraisal of the potential of these drugs to improve motor dysfunction in DS by testing their effects on Ts65Dn mice would not be straightforward, because despite the marked drop in cerebellar volume and GC number and density, and the weaker influence of the tonic-active GABA_A_Rs on electrical properties of GCs described in the current study, changes in sensorimotor performance of Ts65Dn mice have not been consistently observed. Alterations in equilibrium, motor coordination, locomotor activity or gait dynamics have been detected in some studies or in a subset of tests [88,105-111] but not in other studies [26,112-114]. It is not known if the tonic GABA_A_R current is altered in cerebellar GCs of other mouse models of DS.

In summary, we report that GCs in the hypogranular cerebellum of the Ts65Dn mouse model of DS have a smaller tonic GABA_A_R current and weaker expression of the GABA_A_R β3 subunit-gene than GCs in wild-type animals. The smaller tonic current contributes to increased excitability and the firing of APs with increase amplitude and faster kinetics. Similar changes may accompany the decrease in cerebellar GC number in DS and modify cerebellar function.

## Methods

### Animals

Mice were generated by crossing female B6EiC3Sn a/A-Ts(17^16^)65Dn (Ts65Dn) mice, carrying a partial trisomy of chromosome 16 [115], with C57BL/6JEi × C3H/HeSnJ (B6EiC3Sn) F1 males, at the University of Bristol, in accordance with the United Kingdom Animals (Scientific Procedures) Act 1986 and with the University of Bristol Ethical Review Group. Parental generations of all three mice strains were obtained from The Jackson Laboratory (Bar Harbor, Maine, USA). To distinguish trisomic Ts65Dn animals from euploid animals (wild-type, littermates of the trisomic animals), quantitative real-time polymerase chain reaction of tail-tip genomic DNA [116] was used to measure expression of the *App* gene (present in three copies in Ts65Dn and two copies in wild-type animals) relative to expression of the *Apob* gene (present in two copies in both Ts65Dn and wild-type animals; The Jackson Laboratory Protocols) [117].

### Cerebellar slices

Parasagittal slices of cerebellar vermis (200 μm) were prepared from 50 male Ts65Dn mice and 70 male wild-type mice (littermates of Ts65Dn mice) aged P40-60, on a Leica VT1000S vibrating microtome (Leica Microsystems, Nussloch, Germany). Animals were culled in accordance with the United Kingdom Animals (Scientific Procedures) Act 1986 and the University of Bristol Ethical Review Group. Slices were cut in ice-cold sucrose-based solution (in mM: 248 sucrose, 1.3 MgSO_4_, 5 KCl, 2.4 CaCl_2_, 1.2 KH_2_PO_4_, 26 NaHCO_3_, 10 D-glucose, pH 7.4, bubbled with 95% O_2_/5% CO_2_) and stored in standard Krebs-Henseleit solution (in mM: 124 NaCl, 1.3 MgSO_4_, 5 KCl, 2.4 CaCl_2_, 1.2 KH_2_PO_4_, 26 NaHCO_3_, 10 D-glucose, pH 7.4, bubbled with 95% O_2_/5% CO_2_) prior to patch-clamp recording or harvesting of cells.

### Patch-clamp recording and analysis

Individual slices were viewed on a Zeiss FS Axioskop microscope (Carl Zeiss Ltd., Welwyn Garden City, UK). Patch-clamp recordings were made with pipettes (thick-walled borosilicate glass, coated with Sylgard 184, fire-polished) and an Axopatch 200A or 200B amplifier (Axon Instruments, Union City, CA), from slices superfused with standard Krebs-Henseleit solution (~1.5 ml/min) at ~23°C, as described previously [32] and in keeping with previous patch-clamp studies of granule cells at a similar temperature [35,118-121]. Pipettes were filled with, in mM: 135 CsCl, 10 HEPES, 2 MgATP, 10 EGTA (pH 7.2 with TEA-OH) for voltage-clamp recording, or 126 KCH_3_SO_3_, 4 KCl, 10 HEPES, 4 MgATP, 5 EGTA, 4 NaCl, 0.5 CaCl_2_ (pH 7.2 with K-OH) for current-clamp recording, and had resistances of 3.5 – 10 MΩ. Input capacitance measurements of cells recorded in voltage-clamp were taken from amplifier settings used to cancel current transients generated by 5 mV jumps, as in several previous patch-clamp studies of granule cells [32,35,36,119]. Median and quartile values: wild-type, 1.9 (1.5, 2.3) pF, *n* = 58; Ts65Dn, 2.7 (2.0, 3.2) pF, *n* = 38. When cells were recorded in current-clamp, input capacitance was calculated from the time-constant of a single exponential function fitted to the voltage deflection generated by a negative current injection (−10 or −8 pA) [122]. Median and quartile values: wild-type, 3.0 (2.4, 4.1) pF, *n* = 43; Ts65Dn, 3.7 (3.0, 4.0), *n* = 25. GCs of all ages are known to behave as single electrical compartments and the measured input capacitance encompasses capacitances of the soma and dendrites [119].

Current recordings were low-pass filtered by the filter in the Axopatch 200A amplifier (10 kHz, 4 pole Bessel) and then passed through a second filter (2 kHz, 8 pole low-pass Bessel filter, Frequency Devices, Haverhill, MA, USA), or they were filtered by the amplifier-filter alone (2 or 5 kHz), before being digitised on-line at 10 or 25 kHz with a Cambridge Electronic Design (CED) power 1401 A/D interface using Spike2 software (v. 5.13) (CED, Cambridge, UK). Voltage recordings were low-pass filtered (4 pole Bessel filter in the Axopatch 200B amplifier) at 10 kHz and acquired at 62.5 kHz with Signal (v. 3 or 4) or Spike2 software. They were analysed with CED Signal or Spike2 software and with Origin software v.6 or 7 (Microcal, Northampton, MA).

Currents were recorded before and after blockade of GABA_A_Rs by SR95531 (10 μM) at a holding potential of −70 mV. In a few cells, tonic current–voltage relationships were measured by holding the membrane potential at different values between −100 mV and 0 mV for 2–4 s before and during the application of SR95531. The amplitude and variance of the tonic current generated by the activation of GABA_A_Rs by ambient GABA were calculated as the difference between the mean amplitude and variance around the mean of ~5 s periods of digitised data recorded before and during application of SR95531. Sections of data without discrete synaptic currents were chosen for measurement, but if these were rare, multiple periods lacking discrete events adding up to 5 s were measured. Slope conductances at different potentials were calculated from sigmoidal curves fitted to plots of mean current-density against voltage. Plots of SR95531-sensitive variance against SR95531-sensitive mean current measured in many cells were used to derive the mean unitary current of the GABA_A_Rs [45]. Whole-cell and single-channel chord conductances were calculated using the reversal potentials of the mean whole-cell current–voltage curves in wild-type and Ts65Dn GCs. The relative charge transfer during tonic and phasic synaptic currents was calculated from 20–30 s periods of digitised data recorded before and during the application of SR99531 [123]. The decay time course of the phasic currents was determined by fitting a double exponential function or, rarely, a single exponential function to average currents constructed for each recording by aligning the phasic currents on their rising phase (CED Spike 2 software). The zero time point was defined as the time at the peak of the current. The weighted mean time constant was calculated as A_1_.τ_1_ + A_2_.τ_2_, where A_1_ and A_2_ are the fractional amplitudes of the fast and slow components, and τ_1_ and τ_2_ are the time constants of the two components.

Membrane potentials recorded in current-clamp were corrected for a calculated junction potential of 8.8 mV. AP parameters were measured for the first three APs elicited near rheobase (current injection threshold) using Signal or a supplementary Signal script that generated ‘phase-plane plots’ for the measurement of voltage threshold and maximum rates of rise and fall (Steven Clifford, CED), and then averaged. AP height was measured between the peak and the afterhyperpolarisation [124]. Increments in the size of currents injected result in unequal increments in current-density (pA/pF) in different cells, because of cell-to-cell variation in input capacitance. To enable averaging of plots of voltage or AP frequency against current-density, the plot for each cell was interpolated using equally-spaced points (0.5 or 0.1 pA/pF interval) and interpolated values were averaged.

Stock solutions were made of the following drugs: SR95531 (10 mM in filtered Milli-Q water, Tocris Bioscience, Bristol, UK or Ascent Scientific, Bristol, UK), THIP (30 mM in filtered Milli-Q water, Tocris Bioscience), furosemide (100 or 300 mM in 100% DMSO, Sigma). They were stored as aliquots at −20°C and added to the external solution when required. The effects of the drugs THIP and furosemide were measured 11–14 mins after application.

### Single-cell reverse transcription real-time PCR

GABA_A_R subunit mRNAs were quantified in individual GCs harvested from cerebellar slices prepared from 18 male Ts65Dn mice and 25 male euploid littermates (wild-type). These animals were similar in age (P42 – 69) to the animals from which slices were prepared for patch-clamp recording (P40-60). The procedures described below are based on methods developed previously [63]. The study reports absolute quantification of mRNAs in individual cells derived from comparison of real-time data to standard curves, rather than relative expression normalised by expression levels of reference genes.

### Cell harvesting

On the day of cell harvesting, pipettes were pulled from thin-walled borosilicate glass capillaries (Harvard Apparatus, Kent, UK) and fire-polished to a tip size of 1.5 – 3 μm. These had been made RNase-free by one rinse with 0.5 M NaOH, two rinses with absolute ethanol, three or four rinses with sterile ultra-pure Elgastat water (0.2 μm filtered) and baking at 160°C for 2 × 4 hours. In order to harvest a single cell, a pipette was filled with 4 μl of ‘RNA-protecting solution’, which contained 15 U RNasin® Plus or RNasin® ribonuclease inhibitor (Promega, N2515 or N2615, Southampton, UK) and 25 mM DTT (Clontech, accompanies PowerScript reverse transcriptase 639501, Saint-Germain-en-Laye, France). The two components were combined on each day of harvesting and stored on ice. The pipette was placed in the bath solution under positive pressure and moved towards the cell of interest. Once the tip was at the cell, the positive pressure was released and gentle suction was applied to the pipette via a mouthpiece and a length of tubing, so as to draw the cell into the pipette. Suction was stopped as soon as the majority of the cell was in the tip. This procedure was observed on a monitor using AxioVision software and an AxioCam HRm camera attached to the Zeiss FS Axioskop microscope (Carl Zeiss Ltd.). Much effort was put into optimising the size of the pipette tips, by iteratively changing puller settings and fire-polishing. (If the tips were too large, it was difficult to prevent the cell and bath solution from rushing far up the pipette. If they were too small, the cell stuck to the outside of the pipette tip.) After the cell was just inside the pipette, the outside of the pipette was inspected for any extraneous tissue that may have become attached. Such material was blown away with a second pipette containing extracellular solution. If the unwanted material could not be removed, the pipette containing the harvested cell was discarded. We found that it was important to maintain the positive pressure on the pipette until it touched the cell so as to prevent entry of bath solution into the pipette.

### Reverse transcription

In order to release mRNA from the cell, the pipette containing the harvested-cell and 4 μl of ‘RNA-protecting solution’ (see above) was quickly positioned inside a thin-walled 0.2 ml PCR tube (sometimes a 0.5 ml tube) containing 2 μl of a solution that included the detergent Nonidet P40 (0.2%, Roche 11754599001, Mannheim, Germany), plus some reagents necessary for reverse transcription (10 mM Tris–HCl pH 8, Ambion Life Technologies, AM9855G, Paisley UK; 25 μM random hexamers, Roche 11034731001; 2.5 mM of each dNTP, Bioline 39025 or 39028, London, UK). The tip was broken by pushing it against the inside wall of the tube, and the pipette contents were expelled into the mixture by applying pressure (12 psi for 100 ms) via a filtered (0.2 μm) tube. This procedure was observed under a dissecting microscope. The tube was placed in a water bath at 65°C for 5 min and then on ice for 5 min. Additional reagents necessary for reverse transcription were then added. Their final concentrations in a total volume of 10 μl were 10 mM DTT (Clontech, comes with PowerScript reverse transcriptase), 2 U RNasin® or RNasin® Plus (Promega, N2515 or N2615), 1× first strand buffer (Clontech, accompanies PowerScript reverse transcriptase). (Taking into account the reagents present in the harvesting-pipette solution, the solution into which the cell was ejected and the added reagents, the final concentrations of the various components in a total volume of 10 μl were 20 mM DTT, 0.04% Nonidet P40, 5 μM random hexamers, 0.5 mM of each dNTP, 2 mM Tris–HCl, 17U Rnasin® or RNasin® Plus). Reverse transcriptase was then added (PowerScript, 0.5 or 0.75 μl, Clontech 639501, concentration undisclosed) and the tube was incubated at 42°C overnight in a thermal cycler (usually a PTC-200 DNA engine, Bio-Rad, Hemel Hempstead, UK). The reaction was stopped at 75°C for 15 minutes. The tube containing the single-cell cDNA was stored at −20°C.

### Single-cell real-time quantitative PCR

For amplification of cDNA encoding a single type of GABA_A_R subunit in a single harvested granule cell, the mix containing single-cell cDNA was combined with a pair of forward and reverse primers (final concentrations, 400 nM, except for the reverse primer for the Gabrg2 gene, which was 300 nM) (OliGold® purified on a reverse-phase cartridge, Eurogentec, Southampton, UK), 25 μl of a SYBR-Green I mix (QuantiTect SYBR Green PCR kit, Qiagen 204143, Crawley, UK) and water to bring the final reaction volume up to 50 μl. The SYBR-Green I mix contained HotStarTaq DNA polymerase, PCR buffer (composed of Tris–HCl, KCl, (NH_4_)_2_SO_4_, MgCl_2_, pH8.7), a dNTP mix, SYBR Green I and the passive reference dye ROX (concentrations undisclosed by supplier except for MgCl_2_). The final concentration of MgCl_2_ was 2.5 mM. The forward and reverse primers were designed to be located in different exons and to amplify multiple splice variants of a gene of interest, as indicated in Table 1, with the aid of BLAST, Primer-BLAST and Beacon Designer software (version 4, PREMIER Biosoft, Palo Alto, CA, USA). To activate the HotStarTaq DNA polymerase, the tube was incubated on a Stratagene Mx3000P qPCR machine at 95°C for 15 min (Agilent Technologies, Stockport, UK). PCR was executed by cycling the temperature 50 times according to the sequence: 95°C, 15 s; 60 or 61°C, 60 s; 72°C 30 s. This was followed by generation of a melt curve between 55°C and 95°C to confirm the amplification of a single product and to check for the occurrence of primer dimers. cDNA encoding only one type of GABA_A_R subunit was measured per cell.

**Table 1:** **Primers for quantification of expression of GABA**_**A**_**R subunit genes in granule cells with real-time PCR**

**Gene**	**NCBI mRNA reference sequence(s)**	**Primers (5′-3′)**	**Position**	**Amplicon length (bp)**
Gabra1	NM_010250.4	F: GAGCAGAAGTTGTCTATGAGTGG	1349, exon 7	164
		R: GTGGAAGTGAGTCGTCATAACC	1512, exon 8	
Gabra6	NM_001099641.1	F: CTACTCTGAAAATGTCAGTCGGATTC	364, exon 2	123
	(variant 1)	R: CCAAAGCTGGTCACATAGATGTCT	486, exon 3	
	NM_008068.2		364, exon 2	123
	(variant 2)		486, exon 3	
Gabrb2	NM_008070.3	F: AGGGGCTACTTTGGGATTTGG	446, exon 2	108
		R: TCTGTCCACCGTCTCTTTAACC	553, exon 3	
Gabrb3	NM_008071.3	F: TTGCGGAGAAGACAGCCAAG	1128, exon 9	100
	(variant 1)	R: TGAACATCCATCGGTGCTAATAGG	1227, exon 10	
	NM_001038701.1		1060, exon 9	100
	(variant 2)		1159, exon 10	
Gabrd	NM_008072.2	F: GCCAGCATTGACCATATCTCAG	324, exon 3	169
		R: CATTCACGATGAAGGTGTCAGG	492, exon 4	
Gabrg2	NM_008073.2	F: CTTACATTCCCTGCACACTCATC	1181, exon 7	140
	(variant 1)	R: AGATTTTCTGGCTATGGTGCTT	1320, exon 8	
	NM_177408.5		822, exon 7	140
	(variant 2)		961, exon 8	

The PCR reaction volume was relatively large (50 μl) so as to dilute components of the reverse-transcription mixture and thus minimise any inhibition they might have on the efficiency of the PCR assay. Reactions were carried out in strips or plates of 0.2 ml tubes sealed with optically-clear caps (Stratagene 410088; 401425; Fisher Scientific FB68750, Loughborough, UK). In order to minimise variation and allow comparison between wild-type and Ts65Dn cells, PCR of cDNA encoding a single type of GABA_A_R subunit was carried out simultaneously on cells from both wild-type and Ts65Dn mice. For each assay on a batch of cells, the PCR reaction and melt analysis were also concurrently carried out on a non-template control (containing water instead of a cell sample) and on a serial-dilution of known numbers of PCR products in duplicate (9 concentrations expressed as a number of single-stranded copies, spanning ~3.5 orders of magnitude), from which a gene-specific standard curve was generated (Table 2). The lowest points on the curves were 4, 6, 9 or 15 single-stranded copies. Copy numbers of GABA_A_R subunit transcripts in each cell were determined as the number of single-stranded copies relative to the regression lines fitted to the standard curves. Detection of a GABA_A_R subunit cDNA in a cell was defined as an estimated copy number of more than 2.

**Table 2:** Properties of standard curves and lack of signal in no-template controls

**Gene**	**Slope**	**Y-intercept**	**Efficiency (%)**	**Regression**	**Cq of no-template controls**
Gabra1	−3.21	41.22	105	0.995	48.9 (primer dimer)
					(Cq for the lowest unknown concentration was 38.3)
	−3.42	41.72	96	0.993	No signal
	−3.19	41.57	106	0.999	No signal
	−3.30	40.65	101	0.998	No signal
Gabra6	−3.39	41.55	97	0.995	No signal
	−3.48	41.59	94	0.987	No signal
	−3.45	41.91	95	0.997	No signal
	−3.39	41.52	97	0.999	No signal
Gabrb2	−3.22	39.62	104	0.998	No signal
	−3.35	39.35	99	0.991	No signal
Gabrb3	−3.44	40.22	95	0.999	No signal
	−3.37	40.99	98	0.999	No signal
Gabrd	−3.11	40.68	110	0.998	No signal
	−3.18	40.44	106	0.999	No signal
	−3.33	39.49	100	0.996	No signal
	−3.33	39.66	100	0.999	No signal
Gabrg2	−3.28	39.03	102	0.999	No signal
	−3.34	39.70	99	0.996	No signal

### Standard curves for quantification of single-cell copy numbers

For construction of standard curves that were run in parallel with cell samples, PCR products were serially-diluted in 1 mM Tris–HCl pH 8 and 1.25 ng/μl sonicated DNA from salmon testes (included as a DNA carrier, incubated before use at 95°C for 10 min to inactivate potential DNases, Sigma D9156). Each reaction was carried out in duplicate in a volume of 50 μl, containing the same concentrations of primers and SYBR-Green mix as the reactions on single-cell cDNA. So as to mimic the environment in which single-cell cDNA was amplified, the reaction mix was supplemented with reagents used during cell harvesting and reverse transcription (2 μl of the nucleotide/detergent solution (final concentration: 0.4 mM Tris–HCl, 0.1 mM each dNTP, 0.008% Nonidet P40), 2 μl of 5× first strand buffer (final concentration: 0.2×) and 4 mM DTT). A constant threshold fluorescence value was selected from the exponential phase of PCR and the cycle number at this threshold (the quantification cycle, Cq) was plotted against the log of the number of copies in each serial dilution. The parameters of the standard curves are shown in Table 2.

### Generation of PCR products for standard curves

PCR products used to generate gene-specific standard curves that were run alongside cell-samples, were made by conventional PCR of cDNA that we reverse-transcribed from RNA isolated from wild-type mouse cerebellar vermis (see below), using the forward and reverse primers listed in Table 1. PCR reactions were carried out in a volume of 50 μl, which contained 0.5 U Biotaq Red DNA polymerase (Bioline 21038), NH_4_ buffer (composed of 67 mM Tris–HCl (pH 8.7), 16 mM (NH_4_)_2_SO_4_, 10 mM KCl), 2 mM MgCl_2_ (accompanies Biotaq), 0.2 mM each dNTP; 200 nM each primer (Eurogentec); 625 pg or 10 ng mouse cerebellar cDNA. The generation of a single product of expected size was verified by agarose gel electrophoresis. Each amplicon was extracted from an agarose gel (Qiaex II, Qiagen 20021) and the concentration of the product recovered was quantified by running an aliquot on an agarose gel, measuring the intensity of the band (Kodak EDAS 290 imaging system, Carestream, CT) and comparing this intensity to the intensities of quantitative ladders run on the same gel (EZ Load precision Molecular Mass ruler, Bio-Rad 170–8356, Hemel Hempstead, UK). Concentrations were converted to numbers of moles (Promega BioMath calculator; http://www.promega.com/biomath/Default.htm) and then to numbers of double-stranded DNA molecules using the Avogadro constant. Quantified PCR products were stored at −20°C at two concentrations (10^8^ and 10^6^ double-stranded copies/μl) in low-binding 0.5 ml tubes, precoated with 0.1 mg/ml BSA (New England BioLabs, B9001S) in 10 mM Tris–HCl (pH 8, Ambion, Life Techonolgies, Paisley, UK) to prevent binding of the amplicons to the tubes.

### Cerebellar cDNA and initial optimisation of real-time PCR

Prior to amplification of single-cell cDNA with SYBR-Green I, reaction conditions for real-time PCR were optimised by amplification of adult mouse cerebellar cDNA that was made and quantified using standard protocols. Briefly, total RNA was extracted from cerebellar vermis (frozen in liquid nitrogen immediately upon dissection and stored at −80°C) of wild-type mice using TRIzol® Reagent (Invitrogen 15596–026, Life Technologies, Paisley UK), quantified with the Quant-iT RNA Assay Kit (Invitrogen Q32852) on the Stratagene Mx3000P machine and stored at −80°C. The integrity of the RNA was assessed on a denaturing formaldehyde agarose gel. Samples of RNA (1 μg) were treated with DNase I (Ambion AM1906) and reverse-transcribed at 42°C with oligodT primers (Ambion AM5730G) and Powerscript reverse transcriptase (Clontech, as above). The cDNA was purified, concentrated (QIAEX II resin, Qiagen) and quantified at 260 nm (GeneQuant RNA/DNA calculator (Amersham Biosciences, Little Chalfont, UK). PCR reactions (SYBR-Green I) were run in triplicate on 5-fold serial dilutions of cerebellar cDNA covering a range of 128 – 80000 double-stranded copies. The specificity of the PCR products was verified with melting curve analysis and agarose gel electrophoresis. Primer concentrations and the annealing temperature were adjusted until the PCR efficiency was above 90% and the coefficient of variation of the replicate Cq values was below 1. The same conditions were then applied to more dilute serial dilutions of PCR products which were expected to correspond more closely to numbers of transcripts in individual cells (9 dilutions spanning ~3.5 orders of magnitude, the lowest points on the curves were 4, 6, 9 or 15 single-stranded copies). The conditions were altered as necessary, before being used in single-cell PCR (see above and Table 2).

### Statistical tests

Statistical tests on electrophysiological data were performed using Origin (v. 6 or 7), GraphPad Prism (v.4, La Jolla, CA) or PASW Statistics (v. 18, IBM SPSS, Portsmouth, UK) and considered significant at *p* < 0.05. PCR data were analysed with MxPro (v.3, Stratagene), Origin, GraphPad Prism, Microsoft Excel and PASW. Differences were examined using the Student’s paired or unpaired *t* test (when normally distributed as assessed by the Shapiro-Wilk test) or the Mann–Whitney *U* test (when data were not normally distributed). Slopes of fitted linear regression lines were compared using analysis of covariance (ANCOVA). The chi-squared test and Fisher’s exact tests were used to compare the frequency of detection of cDNAs in individual cells. Kruskal Wallis non-parametric ANOVA was used to compare the numbers of different cDNAs. Data are summarized as mean ± standard error of the mean (SEM) or median and quartile values (in parentheses), with *n* denoting numbers of cells.

## Abbreviations

AP: Action potential; DS: Down’s syndrome; GC: Granule cell; GABAAR: GABA type A receptor; P: Postnatal; THIP: 4,5,6,7-tetrahydroisoxazolo [4,5-c] pyridine-3-ol.

## Competing interests

The authors declare that they have no competing interests.

## Authors’ contributions

MS designed and optimised primers and real-time PCR assays, performed single-cell reverse transcription and real-time PCR and analysed the results. RD optimised real-time PCR assays, carried out single-cell reverse transcription and real-time PCR and analysed the results. CG established and supervised genotyping by real-time PCR and helped draft the manuscript. MU conceived the study, made the electrophysiological recordings, harvested cells, analysed electrophysiological and PCR data and wrote the manuscript. All authors critically revised the manuscript and approved the final version.
